# Mosaic tetrasomy 9p: a mendelian interferonopathy associated with pediatric-onset overlap myositis

**DOI:** 10.1186/1546-0096-13-S1-P140

**Published:** 2015-09-28

**Authors:** M-L Frémond, C Gitiaux, D Bonnet, T Guiddir, Y Crow, L de Ponthual, B Bader-Meunier

**Affiliations:** 1 Hôpital Necker, Paris, France; 2 Hôpital Jean Verdier, Bondy, France; 3 Institut Imagine, Paris, France

## Background and objectives

Pediatric-onset inflammatory myositis (IM) and systemic lupus erythematosus (SLE) are rare inflammatory diseases. They result from the complex interaction between genetic and environmental factors. An increasing number of Mendelian conditions predisposing to the development of SLE have been recently identified. They mostly include monogenic conditions, especially type I interferonopathies, associated with an up-regulation of type I interferon (IFN), a key cytokine in SLE and some IM pathogenesis.

## Methods

Report on a pediatric-onset overlap myositis in a 6-year-old girl who carries mosaic tetrasomy 9p.

## Results

The patient presented with myositis overlapping with lupus-like features. Myositis was characterized by a proximal muscular weakness and HLA class I antigens myofiber overexpression on muscle biopsy. Lupus-like manifestations consisted in pericarditis, pleuritis, and positive ANA and anti-SSA antibodies. Complete remission was achieved with three pulses of methylprednisolone, followed by an oral course of steroids in combination with mycophenolate mofetyl. Analysis of tetrasomy 9p showed mosaic tetrasomy in 9p24.3p12 region, including the type I IFN cluster, and increased expression of interferon (IFN)-regulated genes was found.

## Conclusion

These data suggest that mosaic tetrasomy 9p isa new monogenic interferonopathy predisposing to inflammatory myositis and lupus-like features. Therefore, unexplained inflammatory muscle or other organ involvement in patients carrying mosaic tetrasomy of the type IFN cluster of chromosome 9p should lead to the search for IM and/or lupus-like disease, and karyotype should be performed in SLE or IM patients with mental retardation.

